# Magnetically actuatable 3D-printed endoscopic microsystems

**DOI:** 10.1038/s44172-025-00403-8

**Published:** 2025-04-09

**Authors:** Florian Rothermel, Andrea Toulouse, Simon Thiele, Chris Jung, Johannes Drozella, Robert Steinhoff, Harald Giessen, Alois M. Herkommer

**Affiliations:** 1 Institute of Applied Optics (ITO), University of Stuttgart, Stuttgart, Germany; 2 Research Center SCoPE, University of Stuttgart, Stuttgart, Germany; 3 Printoptix GmbH, Stuttgart, Germany; 4 Mikrop AG, Wittenbach, Switzerland; 5 4th Physics Institute, University of Stuttgart, Stuttgart, Germany

**Keywords:** Imaging and sensing, Integrated optics, Mechanical engineering, Endoscopy

## Abstract

In endoscopy, there is a crucial demand for compact system designs to allow for imaging in narrow spaces and reduce the risk of damage during endoscopic procedures. Enhanced functionality of lensed endoscopes can be realized by integrating actuatable imaging systems with flexible fiber bundles. Conventionally fabricated actuatable endoscopes are, however, limited in their miniaturization capability, typically resulting in system diameters greater than 1 mm. In this work, we present highly compact magnetically actuatable 3D-printed and endoscopically integrated microsystems that are fabricated on the end-facet of imaging fiber bundles using two-photon polymerization. Electromagnetic microcoils affixed to the fiber bundles are utilized to stimulate embedded polymer-magnets to achieve axial, lateral, or rotatory displacement of microoptical elements leading to zooming, resolution enhancement, and increased field of view capabilities. All demonstrated systems achieve overall system diameters well below 900 µm, marking a distinct advancement in the miniaturization of actuatable endoscopic devices. This work demonstrates the feasibility of integrating highly functional and compact optical systems within endoscopes, unlocking new potential for their application in diverse fields, for example in minimally invasive (“keyhole”) surgery or intravascular imaging.

## Abstract (web summary)

Rothermel and colleagues present three endoscopically implemented 3D-printed magnetically actuatable micro-optical systems. Active features such as zoom, fiber bundle resolution enhancement and field-of-view shifting were achieved with submillimeter system diameters.

## Introduction

Endoscopic imaging plays a crucial role in medical diagnostics, allowing visualization of internal organs and tissues in minimally invasive or non-invasive procedures^1^. Over the years, continuous efforts have been made to further enhance endoscopes in terms of imaging quality and resolution, as well as accessibility for operators by offering multi-modal operation, such as combining wide-field imaging with subcellular level endomicroscopy^2^. This goes along with a drive to miniaturize endoscopic systems, reducing the risk of trauma and enabling access to confined anatomical areas inside the human body. In that regard, lensless fiber endoscopes using multimode fibers or imaging fiber bundles with attainable outer system diameters of some 100 µm are, to date, unrivaled^3–8^. Depending on the method, they are, however, limited by the requirements of coherent illumination, short working distance, and sensitivity to fiber bending. On the other hand, conventional imaging fiber bundle endoscopes with e.g., gradient-index (GRIN) lenses^9^ or assembled microlens objectives^10^ are more robust but limited in their applicability, as field of view, resolution, and focus cannot be actively adapted.

An approach to increasing the functionality of lensed endoscopes is the implementation of actuated optical components at the distal end of optical fibers or fiber bundles^11–13^. This is typically implemented for scanning applications using electrostatic and electrothermal micro-electro-mechanical systems (MEMS)^14–19^ or piezo actuators^20–27^ to sample the object. Furthermore, endoscopes with tunable optical components have been developed, enabling the control of the focal length^28,29^. A major drawback associated with these conventionally fabricated endoscopic systems featuring actuatable components is their limited miniaturization capability. Optical components, actuators, and mechanical mounting lead to bulky designs with outer diameters considerably greater than 1 mm, which restrict their application in narrow spaces. In addition, the fabrication of these systems requires tedious and error-prone microassembly.

In recent years, 3D-printed micro-optics fabricated by two-photon polymerization (2PP) have enabled ultracompact endoscopic systems^30^. A key feature of this method is the direct fabrication of monolithic imaging systems on the end facet of imaging fiber bundles^31,32^ or single-mode fibers, for example for point-wise imaging^33^, endoscopic OCT^34^, or optical tweezers^35,36^ without the need for microassembly. In contrast to conventional manufacturing methods, it is possible to produce complex free-form shaped optical elements and systems. However, actuatable 3D-printed endoscopic microsystems have not yet been demonstrated. To date, actuation of 3D-printed microstructures is mostly accomplished by the use of stimuli-responsive materials, which are directly incorporated into the 2PP fabrication procedure, commonly referred to as 4D-printing^37^. Optical surface quality, however, has not been reported for these special resins, in contrast to conventional photoresists^38^. Therefore, a multimaterial procedure is necessary. Aside from this, many 4D-printing procedures require specialized setups, which are often not designed for fabrication on fiber tips.

**Fig. 1:**
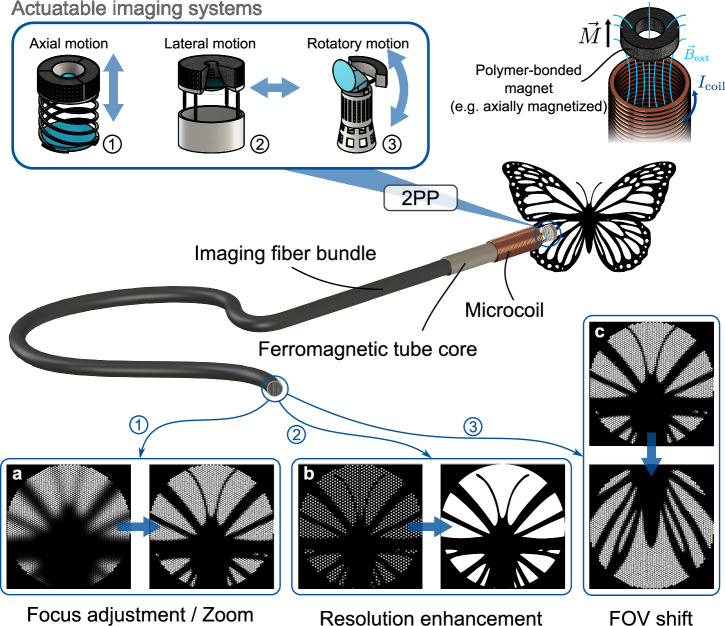
Schematic overview of the herein presented 3D-printed magnetically actuatable endoscopic imaging systems. These enable (**a**) focus adjustment and zoom through axial motion, (**b**) resolution enhancement of fiber bundle images through lateral motion, and (**c**) field of view (FOV) shifts through rotatory motion.

We recently reported the concept and implementation of magnetically actuatable 3D-printed microstructures fabricated on flat substrates with external magnetic stimulation^39,40^. Furthermore, a magnetic microactuator based on this method enabling the bistable positioning of a microlens to switch between high and low numerical apertures has recently been demonstrated^41^. In the work presented herein, we integrated our magnetic stimulation and actuation concepts on the tip of imaging fiber bundles, to facilitate an approach to actuatable endoscopic imaging with unmet diameters in the submillimeter region. We present three 3D-printed microsystems with different degrees of freedom of mechanical motion (Fig. 1). We showcase a system with axial actuation for focus adjustment and zoom capability (Fig. 1a), lateral actuation to enhance the resolution in pixelated fiber bundle images via imageshifts (Fig. 1b), and rotational actuation of a pivotable prism to shift and extend the field-of-view (FOV) (Fig. 1c). Monolithic models, consisting of mechanical, optical, and microfluidic components, are directly printed via 2PP on the facet of an imaging fiber bundle. Within a post-processing step, the microfluidic reservoir is filled with a composite of magnetic NdFeB-microparticles and epoxy resin, which is magnetized after curing to yield a polymer-bonded magnet, embedded in the microsystem. In our endoscopically integrated approach, actuation is facilitated by the implementation of an electromagnetic microcoil directly at the fiber tip. This microcoil is wound around a ferromagnetic tube core encasing the fiber bundle, allowing for extremely compact actuatable endoscopic arrangements with overall system diameters below 900 µm. The applications presented herein demonstrate the potential of this approach, offering new possibilities in scientific fields such as medicine and biology, where precise imaging and control in confined spaces are essential.

## Results

### Axially actuatable system for focus adjustment and zoom

In endoscopic applications, switching from wide-field observation for navigation and location of a region of interest (ROI) to a higher resolution for detailed examination has proven to facilitate diagnostic accuracy, especially in the gastrointestinal tract^42,43^. Customarily, this is often accomplished by combining different types of endoscopes, resulting in bulky system designs that prohibit their use for anatomically challenging scenarios, such as examination of the mucosa of the small intestine or pediatric endoscopy. Therefore, zooming-capability presents an elegant solution that can be realized by axially actuatable lens components. Axial displacement of lenses is also required for refocusing, allowing sharp images to be obtained without repositioning the entire endoscope. This is particularly interesting for endomicroscopic systems, which usually include a small depth-of-focus and therefore require accurate positioning.

**Fig. 2:**
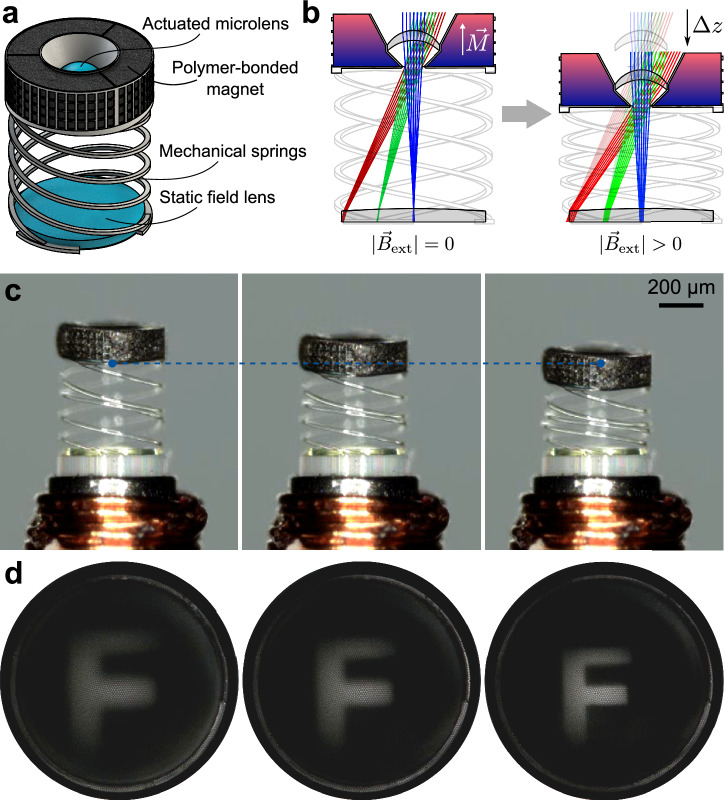
Axially actuatable imaging system for focus adjustment and zoom. **a** Model of the axially actuatable microoptical system. Optical components are colored blue. **b** Concept of the actuation and the optical design layout. The polymer-bonded magnet is magnetized along the optical axis of the imaging system. Application of a rotationally symmetric external magnetic field will result in a shift ∆*z* of the microlens along the optical axis. **c** Microsystem during actuation with the original (left), maximal displaced magnet (right) and in between (center). The blue dashed line marks the same point of the microstructure in each frame. **d** Corresponding images of the letter “F” at the proximal fiber facet. A change in image size can be observed.

A schematic model of a microsystem incorporating an axially actuatable microlens is shown in Fig. 2a. The microlens in the center of a polymer-bonded magnet is mechanically flexibly mounted by three helical springs, allowing its displacement and retraction to its original position. Details on the mechanical design of the springs are given within our previous study^39^. The microfluidically fillable reservoir, which constitutes the polymer-bonded magnet, is separated into three equally sized segments, which are filled separately. Thus, it is ensured that the magnetic particles inside the composite are distributed homogeneously around the optical axis, allowing for a uniform axial motion of the microlens during actuation. The microsystem has an outer diameter of 500 µm and height of 640 µm. It was fabricated on the end-facet of an imaging fiber bundle with a diameter of 500 µm, which, together with the attached ferromagnetic tube core and the two-layered microcoil, sums up to an overall system diameter of approximately 810 µm (Fig. 2c).

The axial displacement of a microlens singlet allows it to either refocus, or achieve moderate zooms in a hyperfocal arrangement. Here, we implemented a zoom system with a high F-number to achieve an enlarged depth of focus and, thus, an adequately focused image for the entire range of motion (Fig. 2b). The optical design was adjusted for the displacement of the microlens singlet of up to 120 µm from its original position towards the image plane. We chose a small aperture diameter of 50 µm at a focal length of 0.63 mm resulting in an F-number of 8.5. The reservoir shape was designed such that the aperture is created by the non-transparent magnetic composite in the final structure. The optimization of the imaging system targeted for a zoom factor of 1/1.4× between the 120 µm displaced singlet position and its origin. The final imaging system consists of the actuated microlens singlet with a back aperture and a static field lens directly in front of the image plane, that is, the fiber bundle facet (Fig. 2b). Both lenses are aspheric to 8th order. The field lens is mainly required to reduce the incident chief ray angles of the outer fields on the image plane, such that they are within the acceptance angle of the fiber bundle given by its numerical aperture (NA) across the full actuation range. Given the criteria of incidence angle and depth of focus, a theoretical zoom factor of 1/1.36× could ultimately be achieved through optimization.

The polymer-bonded magnet is magnetized in the direction along the optical axis of the imaging system, resulting in its axial displacement when the microcoil is activated, i.e., $|{\vec{B}}_{{ext}}| > 0$. By controlling the magnetic flux density via the coil current, the microlens can be continuously positioned along the optical axis. Its original position is restored by the mechanical springs when the microcoil current is switched off. The microlens can be displaced in both directions by changing the coil current polarity, leading to attractive or repulsive forces.

For the experimental investigation of the axially actuatable system, we imaged a negative target of the letter “F” with a diffuse backside illumination (Fig. 2d). The letter had a height of 12 mm and was placed at a distance of approximately 30 mm in front of the endoscope, well beyond the hyperfocal distance. A triangular current pulse ranging from 0 to 160 mA with a rise and fall time of 5 s each, was applied to the microcoil. The microsystem and the image at the proximal end of the imaging fiber were observed with a digital microscope during the actuation (Fig. 2c, d). It should be noted that the microsystem and image were not simultaneously observed. A video of the axially actuated system and the corresponding image are provided in the Supplementary Information, Supplementary Movie S1. From the blue dashed line, marking the initial point of the microsystem in each frame, a maximal axial displacement of approximately 120 µm can be estimated, which corresponds to the configuration in the optical design. The corresponding images of the letter “F” clearly demonstrate the intended zoom effect. Estimating from the sizes of the image in the original and maximally displaced states, the zoom factor is approximately 1/1.3×, which is in good agreement with the design.

### Laterally actuatable system for resolution enhancement

Endomicroscopy featuring imaging fiber bundles was already introduced in the early 1990s^44^ and has since established as important tool for in-vivo histological imaging in many different clinical applications^45^. Due to their enhanced miniaturization capability, 3D-printed endomicroscopic systems could potentially be applied to hardly accessible organs of the gastro-intestinal or respiratory tracts and yield highly-resolved, diffraction limited images^32^. The resolution is however limited by the honey-comb image pixelation caused by the fiber bundles, which is a well-known issue. To overcome this limitation, an often used approach is to reconstruct the image from multiple, laterally shifted images^20,21,46^. In a single image, information is missing due to the cladding of the fiber bundle. By slight lateral shifts of the image, this missing information can be acquired, and a non-pixelated image can be reconstructed through algorithmic processing of the image stack. In this manner, a resolution enhancement can be achieved.

**Fig. 3:**
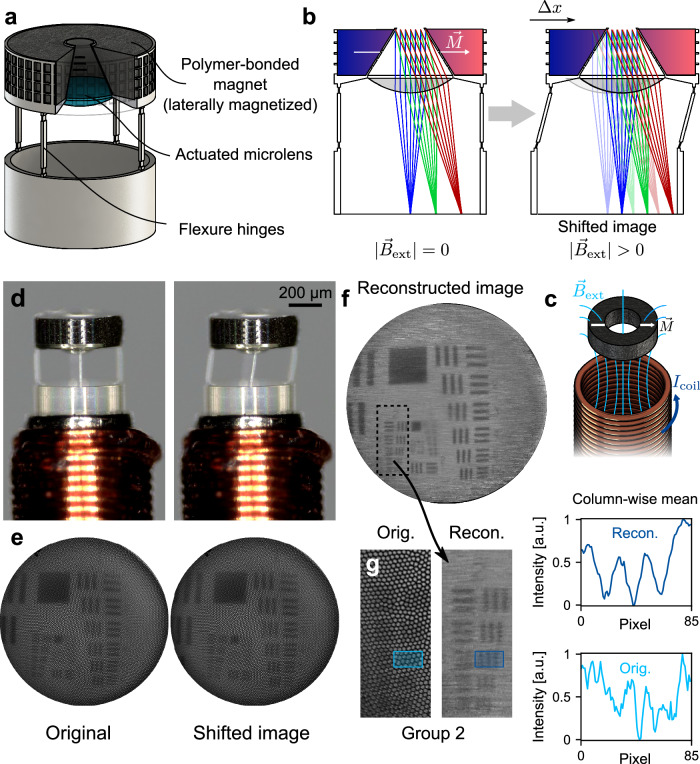
Laterally actuatable imaging system to enhance the resolution of the pixelated images obtained from fiber bundles. **a** Model of the laterally actuatable micro optical system. The cut reveals the actuated micro lens colored in blue. **b** Concept of the actuation and the optical design layout. The polymer-bonded magnet is magnetized perpendicular to the optical axis of the imaging system. Application of an external magnetic field will result in a lateral shift ∆x of the microlens, and thus, the image. **c** Concept of the laterally magnetized polymer-bonded magnet within the magnetic field of the microcoil. **d** The microsystem during actuation with the original position on the left and the laterally displaced lens on the right side. **e** The corresponding images of a USAF target obtained during actuation. **f** Reconstructed image obtained from 60 images. **g** Comparison of the original and reconstructed image of the USAF target group 2. On the right side, the column-wise mean intensity of the marked ROI (group 2–element 3) of the reconstructed (top, dark blue) and original (bottom, light blue) image is displayed.

Image shifts require lateral displacement of the imaging system with respect to the object, which, in a controlled manner, can be achieved by the implementation of a laterally actuatable optomechanical mount. Using the concept of magnetic actuation, we realized a 3D-printed endoscopic microsystem capable of this feature. Figure 3a shows the schematic model of this system. The polymer-bonded magnet and the microlens are mounted with four rectangular flexure hinges on top of a solid ring, enabling lateral displacement of the microlens through external magnetic stimulation. This leads to a shift of the image (Fig. 3b). The optical design of the imaging system consists of an aspheric plano-convex microlens with a front aperture with a diameter of 100 µm. The optimized design yields diffraction limited resolution in the center with a theoretical Rayleigh resolution limit of 122 µm in the object space at an object distance of 20 mm and a wavelength of 500 nm. The resolution slightly decreases with increasing field angle. During actuation, the image is laterally shifted by several micrometers, further increasing the outer field angle. For the optimization, we assumed a shift of up to 20 µm. It is to note here, that by controlling the polarity of the coil current, it is possible to achieve both positive and negative shifts in one axis. Due to the nature of the magnetic actuation, it is, however, not possible to shift in the direction of the opposing lateral axis, resulting in an increased resolution enhancement primarily in one direction. This, however, only poses a notable problem if the imaged structures are perfectly parallel to the translation axis, which in practice, particularly in biomedical applications, can be considered as extraordinarily rare.

Regarding the mechanical design of the flexure hinges, the lateral displacement ${u}_{x}$ of the structure in the intended direction can be approximated by four parallel leaf springs according to the bending beam model:

1 $${u}_{x}=\frac{{F}_{{mag}}{L}^{3}}{4{b}_{S}{t}_{S}^{3}E}$$

Here, ${F}_{{mag}}$ is the magnetic force exerted onto the polymer magnet which typically is within a range of 0 to 100 µN (see Supplementary Fig. S4, Supplementary Information) according to numerical simulations. The Young’s modulus $E$ of the used photoresist IP-S (see Material & Methods) can be assumed to be in a range of 3 to 5 GPa. A fully linear elastic behavior is not to be expected in reality, however. The flexure hinges each have a length $L$, width ${b}_{S}$, and thickness ${t}_{s}$ in the final design of 220, 20 and 10 µm, respectively. They incorporate two rectangular notches 160 µm apart, where the thickness is reduced to 4 µm over a length of 20 µm, leading to compliance of the flexure hinge to lateral forces perpendicular to its wider side. In contrast, the lateral motion perpendicular to the thinner side as well as the axial motion are constrained. Therefore, the polymer-bonded magnet must be laterally magnetized in the compliant direction of the flexure hinges (Fig. 3c). Torque and lateral forces are exerted on the laterally magnetized body by the external magnetic field of the microcoil, ultimately leading to a lateral shift as prescribed by the flexure hinges. The final mechanical design was developed by means of numerical finite element simulations (see Supplementary Figs. S5 and S6, Supplementary Information) with the goal to achieve a lateral displacement of at least 8 µm, corresponding to an image shift of approximately one core-to-core pitch of the imaging fiber bundle. Its viability was lastly verified through experimental investigation.

The laterally actuatable microsystem has a total height of 641 µm and an outer diameter of 500 µm. It was fabricated on the tip of an imaging fiber bundle with a diameter of 500 µm. Therefore, the overall diameter of the endoscopic system is also at approximately 810 µm (Fig. 3d).

For experimental validation, we imaged a backside-illuminated positive USAF target using the laterally actuatable system. The target was placed at a distance of approximately 20 mm. We applied a triangular current pulse within the range of −180 mA to +180 mA with a duration of 60 s to the microcoil, thus shifting the lens in the positive and negative direction. During actuation, the microsystem and the image at the proximal end were observed and recorded separately using a digital microscope. A video of this experiment displaying the microsystem and the image during actuation, is provided in the Supplementary Movie S2. In Fig. 3d, e, the microsystem and the corresponding images are presented for the original and maximum displacements in one direction. Controlled motion of the microsystem was achieved, and a lateral shift of the image was observed. It is important to note that the current pulse duration can be considerably shorter, i.e., rectangular current pulses can be used for actuation. A longer duration, however, leads to an increased range of motion as the displacement increases with time under load owing to the viscoelastic behavior of the material.

The single images show that the fiber bundle cores ultimately limit the resolution of the image across the entire field (Supplementary Fig. S2, Supplementary Information). From the video recorded during the image shifting, 60 frames were obtained and processed using a reconstruction algorithm^46^. The reconstructed image is shown in Fig. 3f. Resolution enhancement is visually perceivable compared with the original image. Reconstruction artifacts, that is, noise along the shifting direction, which is especially notable in the outer image area, indicate an error of the shift estimation by the algorithm. This is likely caused by the limited contrast in combination with several image shifts of only a few pixels. Despite that, an evaluation of the resolution of the reconstructed and original image proves the functionality of the approach. As indicated in Fig. 3g, we compared the imaging of group 2, element 3, which corresponds to a line pitch of 198 µm, within the same ROI of both images. The column-wise mean intensity inside the ROI was computed. For the reconstructed image, the three lines are clearly distinguishable, whereas a static system (original image) would fail to resolve these lines.

### Rotatory actuatable system for FOV shifting

Conventional endoscopes with static lens systems have a fixed FOV, which poses a limitation to the situational awareness of the operator, e.g., during minimally invasive surgeries, since the viewing angle has to be adjusted by repositioning or withdrawing and exchanging the optics. Commercially available products, such as the ENDOCAMELEON (Karl Storz GmbH, Germany) or the ENDOEYE FLEX 3D (Olympus Corp., Japan) besides other patent applications^47–50^ incorporate pivotable prisms that allow for varying the viewing direction. Another system presented by Zuo et al.^51^ presented a system for flexible adjustment of the FOV and scanning with fiber bundle endomicroscopes. As these devices incorporate bulky mechanical parts, employment in narrow spaces, such as for observation during minimally invasive procedures inside the sinus cavities is limited. It could therefore potentially be advantageous to implement magnetically actuatable, pivotable 3D-printed optical systems that allow the FOV to be shifted, as the accessibility of challenging areas can be enhanced.

**Fig. 4:**
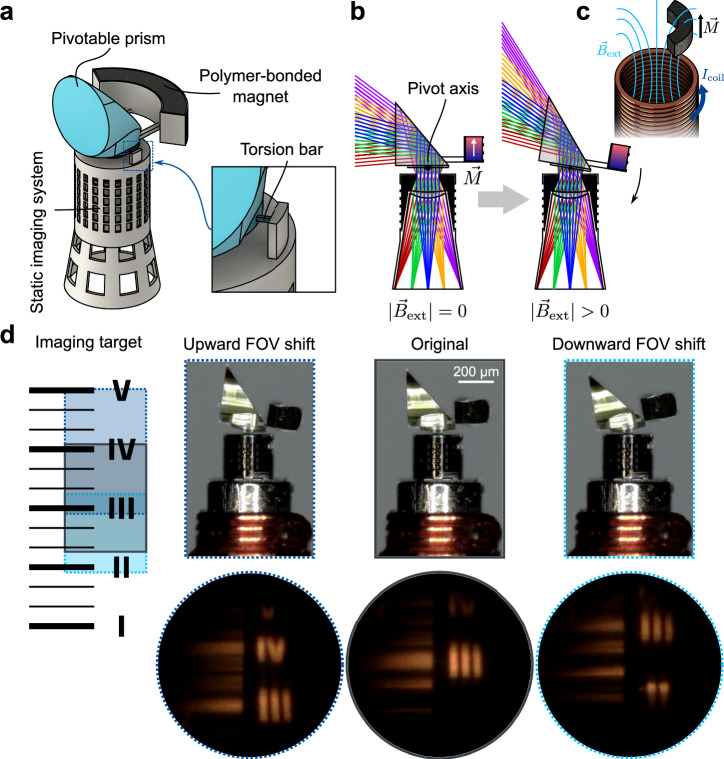
Rotatory actuation of a pivotable prism for FOV shifting of an imaging system. **a** Model of the micro optical system with pivotable prism (colored in blue) that is mounted by two symmetrically arranged torsion bars, as shown by the zoomed-in view. The static imaging system consists of a singlet lens with front aperture, which is realized through an ink-filled chamber in the mechanical mounting. **b** Concept of the actuation and the optical design layout. The polymer-bonded magnet is magnetized along the optical axis of the imaging system. Application of an external magnetic field results in a rotation of the prism around its pivot point, hence shifting the observed area. **c** Concept of the off-centered and axially magnetized polymer-bonded magnet within the magnetic field of the microcoil. **d** Imaging and actuation experiment. The imaging target shown on the left side was observed. The marked areas indicate the observable regions in the original (gray), upward shifted (dark blue) and downward shifted (light blue) case. In the center, the actuated microsystem along with the corresponding images on the right side is displayed.

We developed a side-facing imaging system that incorporates a rotatory actuatable prism (Fig. 4a). The FOV is shifted by pivoting the prism, enabling the operator to observe an enlarged area. In the optical design, a rotation range of the microprism of −10 to +10° was considered for optimization. The static imaging system with a focal length of 0.6 mm and an F-number of 4.2 comprised an aspheric microlens singlet and a front aperture with a diameter of 110 µm (Fig. 4b). It corrects for aberrations caused by the prism, such that a nearly diffraction limited optical performance over the entire FOV and range of rotation could be achieved in the optical design.

The microprism is mechanically mounted by two symmetrically arranged torsion bars with a length of 16 µm. They have a square cross section with a side length of 5 µm. The reservoir containing the polymer-bonded magnet is attached to the backside of the prism. A cavity was integrated into the mounting of the static microlens, which is filled with a non-transparent ink to create the aperture and reduce the loss of contrast due to stray light^52^. The incoming light is redirected by total internal reflection (TIR) at the 50° inclined backside of the microprism and focused by the singlet onto the fiber bundle end facet, as shown in the concept in Fig. 4b. The polymer-bonded magnet is magnetized in the axial direction (Fig. 4c). Therefore, the external magnetic field of the microcoil results in attraction or repulsion, depending on the polarity of the current. Because the magnet is attached off-axis to the microprism, the magnetic force leads to rotatory motion around the pivot axis, which is prescribed by the torsion bars. With the assumption of a linear-elastic behavior of the material, the rotational angle of the prism ${\phi }_{P}$ can be approximated by:

2 $${\phi }_{P}=\frac{{3M}_{t}{L}_{{TB}}}{G{a}_{{TB}}^{4}}$$

The torsional moment ${M}_{t}$ is given by the magnetic force and the lever. $G$ is the shear modulus of the photoresist, ${L}_{{TB}}$ and ${a}_{{TB}}$ are the respective length and square section side length of the torsion bar. The final design was again obtained with support of FEM simulation, predicting a prism rotation of approximately 10° with the assumption of a typical achievable magnetic force of 20 µN. Using the same conditions, Eq. 2 predicts a rotation of approximately 12.5°. By switching off the magnetic field, the prism is rotated back into its original position.

This microsystem was printed onto the tip of an imaging fiber bundle with a diameter of 350 µm. Using the attached electromagnet, an overall system diameter of approximately 660 µm is achieved. The 3D-printed microsystem has a height of 740 µm and maximum outer diameter of 550 µm (Fig. 4d). The tube core of the electromagnet was raised above the fiber bundle facet to cover the lower, transparent part of the 3D-printed system, which also reduces the distance between the microcoil and magnet. Hence, higher magnetic forces can be attained, thereby increasing the actuation sensitivity.

Actuation experiments were conducted again under observation using a digital microscope. Frames of the recorded videos, showing the actuated microsystem and the image at the proximal end of the fiber bundle are displayed in Fig. 4d. The actuation of the microsystem and the corresponding fiber bundle image are shown in Supplementary Movie S3. A custom ruler test target shown on the left side of the figure was observed at a distance of approximately 20 mm. The Roman numerals and thick horizontal lines are 10 mm apart in the original scale. The target was positioned such that the Roman numeral three was in the center of the image in the original FOV of the microsystem. To pivot the prism, a triangular current pulse ranging from −180 to +180 mA with a duration of 20 s was applied to the microcoil. A smooth rotation of the prism can be observed, which leads to an upward and downward shift of the FOV as intended. The microscope images indicate an experimentally achieved downward rotation of −6.9° and an upward rotation of +9.0°. The observable areas are marked on the test target in Fig. 4d with corresponding colors. The areas differ in size due to the oblique viewing angle and furthermore, a violation of the TIR condition of lower field angles, which is especially notable when facing downwards. Nonetheless, the FOV is considerably extended in comparison to the original image by the rotatory actuation of the microprism.

## Discussion

In this study, we presented three ultra-compact endoscopes with different active imaging modalities: zoom, resolution enhancement, and FOV shift through axial, lateral, and rotatory movements, respectively. These systems showcase the versatility of a magnetic actuation method for 3D-printed microoptical systems and their compact endoscopic integration. To the best of our knowledge, such thin lensed fiber bundle endoscopes with diameters <900 µm and active optical features have not yet been demonstrated. Furthermore, our method does not require microassembly, unlike conventionally fabricated actuatable endoscopic devices. However, the herein demonstrated systems are not encapsulated, prohibiting their application in aqueous in- or ex-vivo environments at the current stage. Static 3D-printed endoscopic systems with immersion capability have recently been demonstrated^53^, which could be implemented in similar way for the actuatable devices. An overview and comparison of several endoscopic systems with implemented actuation is shown in Table 1.

**Table. 1:** Comparison of different miniaturized actuatable endoscopic devices

	Size	Actuation method	Optical system	Active features	Fabrication	Encapsulation
Ref.^28^	2 mm	Piezo benders	Tailored and optimized polymer molded elements (asphere/freeform)	Zoom	Microfabrication and -assembly	No
Ref.^14^	1.65 mm	Electrothermal MEMS	GRIN lens	Image scanning for endomicroscropy	Flip-chip bonding and microassembly	Yes
Ref.^29^	7.5 mm	Hydraulic	Tunable spherical liquid lens	Focal length tuning, i.e., zoom	Microfabrication and -assembly	Yes
Ref.^21^	5 mm	Piezo tube	GRIN lens	Two-directional lateral image shift	Microassembly	No
Ref.^23^	2.4 mm	Piezo tube	Tailored GRIN lens with additional glass elements (refractive/diffractive)	Image scanning for multimodal endomicroscopy	Microassembly	Yes
Ref.^19^	2.6 mm	Electrostatic MEMS	Scanning mirror with additional refractive lens system	Wide-field fluorescence image scanning	Microfabrication and -assembly	Yes
This work	660–810 µm	Magnetic	Tailored and optimized 3D-printed polymer elements (asphere/freeform)	Zoom, one-directional lateral image shift, FOV shift	Monolithic 3D-printed system with post-process magnetic functionalization	No

Owing to its easy integration into the fabrication procedure without compromising the advantages of 2PP, our approach advances the capability of 3D-printed microoptical systems in general. Our findings can advance the fabrication of complex optical systems combined with custom-shaped actuated mechanical structures in the future. The presented method allows for both, large deformations as shown by the axially actuatable system, and subtle but mechanically prescribed motions as shown by the laterally and rotatory actuatable systems.

The optical performance of the demonstrated microsystems is comparable to that of the static 3D-printed microoptical imaging systems presented in other studies^30^. Therefore, we conclude that mechanically flexible mounting does not noteably affect the surface quality of the optical components. It is important to note that the imaging systems shown here are highly efficient, yet simple proof-of-concepts, consisting of not more than two optical components. However, optical designs can be further tailored to specific demands for example, the laterally actuatable system can be adjusted for endomicroscopic imaging^32^. For instance, the images obtained by the axially actuatable system (Fig. 2d) indicate that the optical performance is limited by surface deviations of the microlenses, causing aberrations and shifting of the ideal image plane. The image in the maximally displaced case appears to be more focused in comparison with the case without maximum displacement. It was intended that the image would remain focused throughout the entire range of motion under our intended hyperfocal imaging condition. Surface deviations originate from the shrinkage of the photo-cured polymer and can be corrected by means of iterative procedures, which have proven to yield considerably enhanced imaging quality^54,55^. The printing artifacts observed at the TIR-surface (Supplementary Fig. S3, Supplementary Information) of the rotatory actuatable system can, however, not be corrected in this way, but a much higher surface quality is attainable with, e.g., two-photon grayscale lithography (2GL)^56,57^. Additionally, imaging contrast can be improved by implementation of non-transparent hulls^52,58,59^, which require additional post-processing.

An important aspect of 3D-printed actuatable microsystems is the management of hysteresis, which originates from the magnetic properties as well as the viscoelasticity of the polymerized structure. Hysteresis management has been extensively studied in our previous work^39^. Both hysteresis effects can limit the precise positioning, repeatability, and response time of the presented microsystems. In addition, heat generated by the microcoil could further affect and amplify the unwanted viscoelastic behavior. Future studies could therefore aim to establish a feedback control loop to adjust the actuator displacement according to the image information, e.g., contrast or tracking of feature size and shift. Another possibility are additional Fabry-Pérot based sensors to either directly measure the displacement^60^ or indirectly, by tracking changes in the magnetic field^61^ caused by displacement of the polymer-bonded magnet. Distinct cores of the fiber bundle can be used for sensing, thus avoiding an increase in the overall system diameter.

With regard to the axially actuatable system, we observed that the magnet can deviate from axial motion during actuation. The mechanical springs are designed for axial displacement, but they also allow for lateral displacement and tilt if non-symmetric magnetic forces are present. This can be caused by the misalignment of the magnetic field provided by the microcoil, magnetization direction of the polymer-bonded magnet, or magnetic particle distribution across the microfluidic reservoir. These unintended movements can introduce aberrations into the optical system. To counteract this issue, the mechanical design could incorporate additional guiding bars to stabilize the magnet. It is important to manage the friction caused by the sliding mechanism, for which lubrication using ferrofluids^62^ could be an option. If only small axial displacements are required, flexure hinges can be used instead of compression springs.

In conclusion, we demonstrated the feasibility of highly compact magnetically actuatable endoscopic systems and the versatility of their application. We consider this work an impactful advancement in the miniaturization of endoscopes with active optical features. Our work could open the path to a multitude of novel applications in 3D-printed endoscopic microsystems with active components, such as, imaging systems with magnetically actuatable grippers for biopsy. With regard to medical applications, it is also possible to encapsulate these systems while maintaining small outer diameters^34^ and without interfering with the magnetic actuation.

## Materials and methods

### Imaging fiber bundle preparation

The axially and laterally actuatable system were both fabricated on an imaging fiber bundle of type FIGH-10-500N with an outer diameter of 500 µm and the rotatory actuatable system on a FIGH-10-350S (both Fujikura Ldt., Japan) with an outer diameter of 350 µm. Both the fibers contain approximately 10,000 fiber cores. The fiber end facets were stripped and polished.

For the fabrication of the electromagnet, iron-nickel-alloy tube cores (Nippon Tokushukan MFG. Co.,Ltd., Japan) with lengths of 20 mm and wall thicknesses of 100 µm and corresponding inner diameters for the respective fiber bundles were applied. Insulated copper wire with a diameter of 50 µm was wound directly around the tube cores to fabricate the microcoil, using a custom-made winding setup. The microcoil consists of 170 turns in two layers, resulting in a length of approximately 5 mm. Finally, the electromagnet was joined to the fiber bundle.

### Fabrication of the microsystems

All the microsystems were 3D-printed via 2PP using the Photonic Professional GT2 (Nanoscribe GmbH & Co. KG, Germany) directly onto the end facet of the imaging fiber bundles in dip-in configuration. For the process, a 25× objective with an NA of 0.8 and a working distance of 0.8 mm was used with the proprietary photoresist IP-S for all samples. Prior to printing, the mechanical components of the 3D-models were sliced and hatched with 0.2 µm slicing distance and 0.5 µm hatching distance, optical components with 0.1 µm slicing distance and 0.25 µm hatching distance. Furthermore, the monolithic 3D-models were split into several parts (e.g., nine parts for the rotatory actuatable system) that are used to define the order of manufactured elements during the printing procedure. For the writing process, a laser power between 40% and 50% (corresponding to 20–25 mW) at a constant galvo scanning-speed of 100,000 µm s-1 was used. The structures were developed after printing with PGMEA (AZ EBR Solvent, Microchemicals GmbH) for 12 min and rinsed with isopropanol for 2 min.

Following the printing procedure, the reservoir of the respective sample was filled with a composite of NdFeB-microparticles (MQFP-14–12, Magnequench GmbH, Germany) and a two-component epoxy resin (Epox 200 G + Haerter 120 L, DD Composite GmbH, Germany) with a weight mixing ratio of 2:1 (particles to epoxy). After curing, the samples were exposed to a homogeneous magnetic field with a magnetic flux density of > 3 T, using a custom-made magnetization fixture and an industrial pulse-magnetizer (MC2K10, MAGSYS magnet systeme GmbH, Germany). This results in the permanent magnetization of the composite. A more comprehensive description of the fabrication procedure can be found in our previous study^39^.

### Optical and mechanical design

The optical designs for all three microsystems were realized in the raytracing software Zemax OpticStudio 21 and were optimized for spot size, considering the displacement of the actuated micro-optical element by multi-configurations. Furthermore, the incident angles of the chief rays at the image plane, i.e., the fiber facet were constrained by the acceptance angle of the fiber cores. The optical components of the finalized systems were exported as CAD files.

Based on the arranged optical elements, the mechanical structures of the systems were added using the CAD software SOLIDWORKS 2023. The functionality of the mechanical structures was investigated prior to fabrication in mechanical and partially in magnetic finite element simulations^39^ using COMSOL Multiphysics 5.6.

In preliminary studies of the imaging systems, samples were fabricated on glass substrates and imaging tests were conducted to assess the optical performance. Surface deviations of optical elements due to polymer shrinkage cause a defocused image, which was compensated for by either trimming or extending the height of the 3D-model.

### Actuation experiments

To observe the microsystem and images at the proximal fiber ends, a VHX-7000 digital microscope (Keyence, Japan) with a pivotable objective was utilized. With the exception of the USAF target, different imaging test targets were custom-made from cardboard, using a laser cutter. All targets were adhered to a 6 W diffuse areal illumination LED and manually positioned in the FOV of the respective imaging system. The microcoil was connected to the amplified output of an arbitrary waveform generator (MFG-2120MA, Good Will Instruments Co., Ltd., Taiwan). Using a precision potentiometer, the maximum output current was limited to ±180 mA to avoid thermal damage of the microcoil. Between the actuation experiments, the ferromagnetic tube core was demagnetized by applying a decaying sine function pulse, as proposed in our former study^39^. The schematic setup is shown in Supplementary Fig. S1, Supplementary Information.

### Resolution enhancement with the laterally actuatable system

For resolution enhancement, a video of the fiber bundle image was recorded at full resolution with a frame rate of 15 frames per second during the actuation experiment. In order to reduce memory usage during reconstruction, we extracted one frame per second in post-processing and omitted redundant images, which led to an image stack of 60 frames in total. The reconstruction was performed using the Python-based open source fiber bundle reconstruction algorithm PyFiberBundle provided by Hughes^46^.

## Supplementary information

Supplementary Information

Description of Additional Supplementary Files

Supplementary Movie S1

Supplementary Movie S2

Supplementary Movie S3

## Data Availability

The data that support the findings of this study are available from the corresponding authors upon reasonable request.
