# Supplementary material for: Magnetically actuatable 3D-printed endoscopic microsystems
Source: Commun Eng. 2025 Apr 9;4:69. doi: 10.1038/s44172-025-00403-8 (PMC11982310; doi:10.1038/s44172-025-00403-8)
Supplement: Supplementary file 1 — Supplementary Information [file 44172_2025_403_MOESM1_ESM.pdf]

Supplementary information for

Magnetically actuatable 3D-printed endoscopic microsystems

Florian Rothermel[1,2], Andrea Toulouse*[1,2], Simon Thiele[3], Chris Jung[4], Johannes Drozella[1,2], Robert Steinhoff[1,2], Harald Giessen[2,5] and Alois M. Herkommer[1,2]*

Affiliations:

[1]Institute of Applied Optics (ITO), University of Stuttgart, Pfaffenwaldring 9, 70569 Stuttgart, Germany

[2]Research Center SCoPE, University of Stuttgart, Pfaffenwaldring 57, 70569 Stuttgart, Germany

[3]Printoptix GmbH, Nobelstr. 15, 70569 Stuttgart, Germany

[4]Mikrop AG, Industriestr. 22, 9300 Wittenbach, Switzerland

[5]4th Physics Institute, University of Stuttgart, Pfaffenwaldring 57, 70569 Stuttgart, Germany

*Correspondence: rothermel@ito.uni-stuttgart.de, toulouse@ito.uni-stuttgart.de

Content

1. Experimental setup
2. Image comparison of the laterally actuatable system
3. Measurement of the TIR surface of the pivotable prism
4. Design of the flexure hinges
5. Supplementary Movies

1. Experimental setup

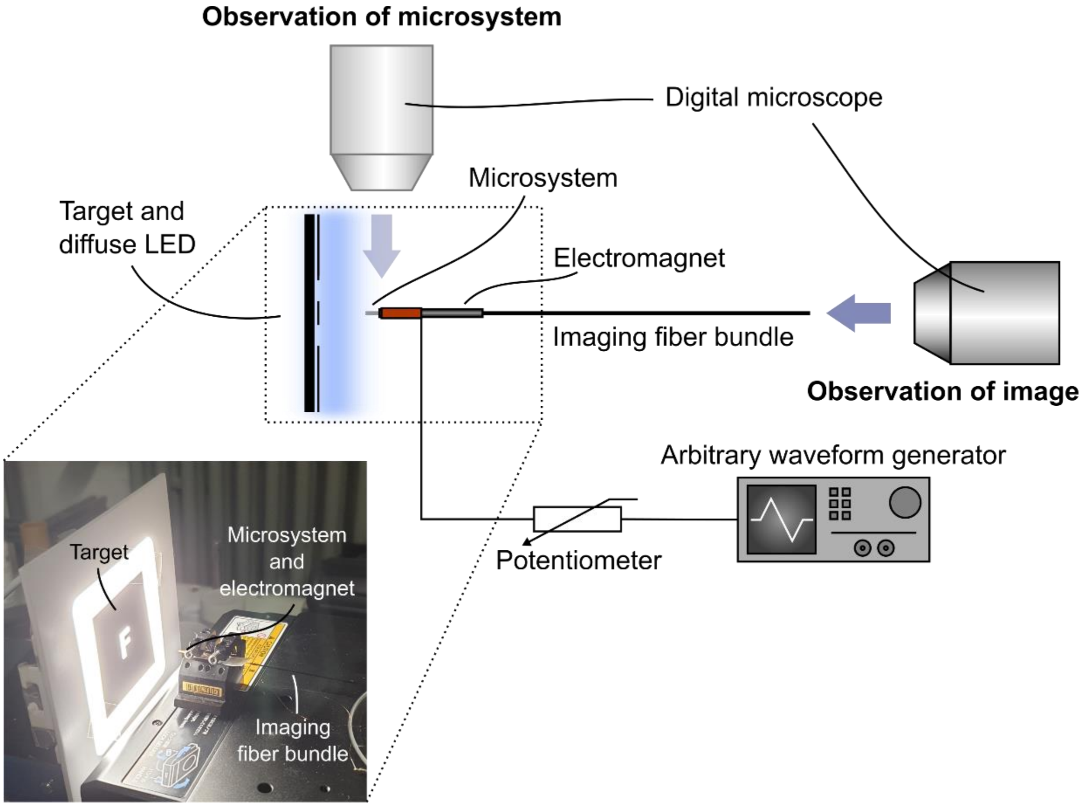

Supplementary Figure S1 Experimental setup for observation of the microsystems and the image at the proximal end of the imaging fiber bundles during actuation. The description of the setup is given in the main text.

2. Image comparison of the laterally actuatable system

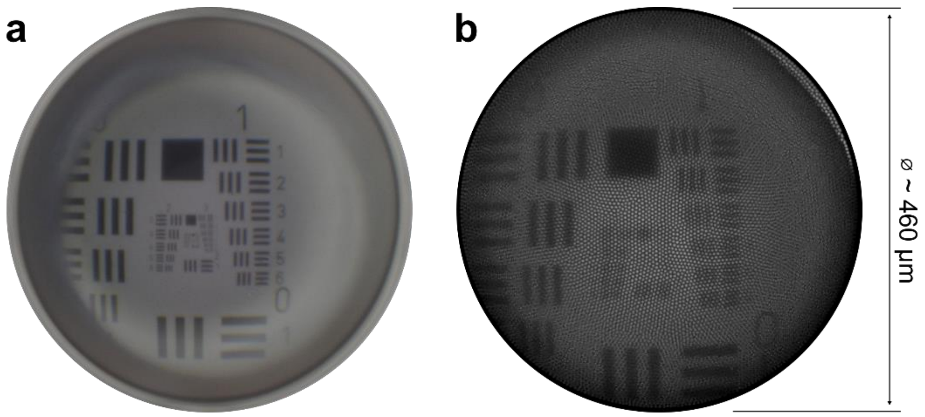

Supplementary Figure S2 Image comparison of the laterally actuatable system for a system printed on a standard glass substrate (a) and printed on an imaging fiber (b). It is visible that the resolution is governed by the pixelation of the fiber cores. It is to note that there was a slight difference in object distance.

3. Measurement of the TIR surface of the pivotable prism

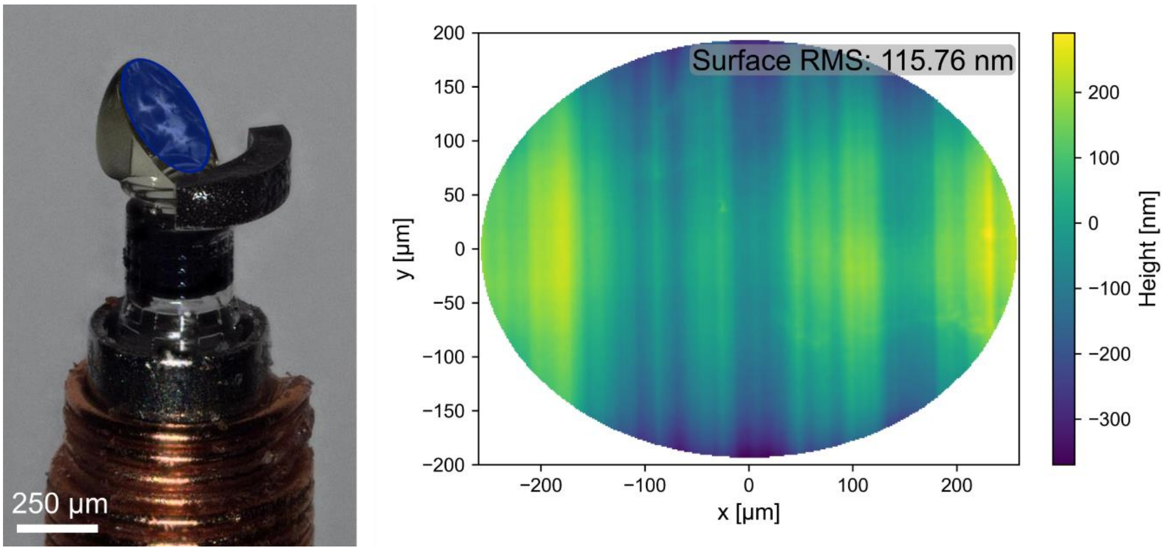

Supplementary Figure S3 Measurement of the TIR surface of the pivotable prism by white light interferometry (Nexview NX2, Zygo, USA). Printing artifacts ("staircase effect") are notable, which cause an RMS surface deviation of 115.76 µm. This leads to aberrations that ultimately limit the optical performance of the FOV shifting system.

4. Design of the flexure hinges

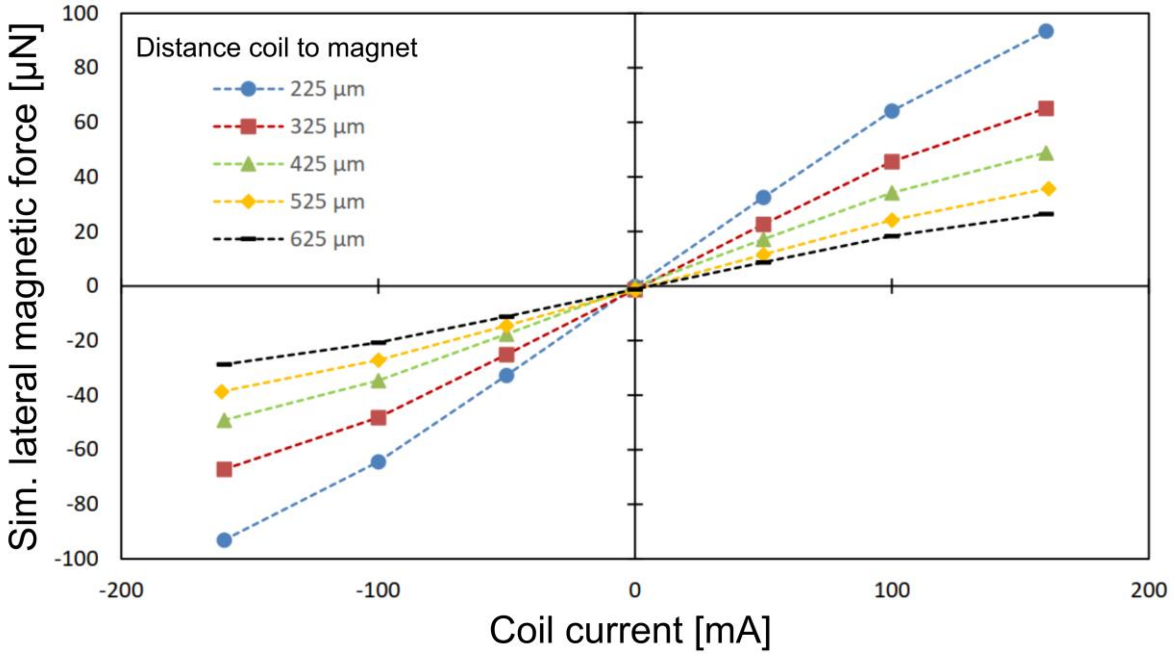

Supplementary Figure S4 Simulated lateral magnetic force exerted onto the polymer magnet depending on the coil current (-160 to 160 mA) and on the edge-to-edge distance between the polymer magnet and the microcoil. A magnetic 3D FEM simulation was used. The magnet has a remanence of 215 mT and is diametrically magnetized. For the FeNi-tube, a built-in model of the anhysteretic B-H curve was used for the magnetic behavior of the material.

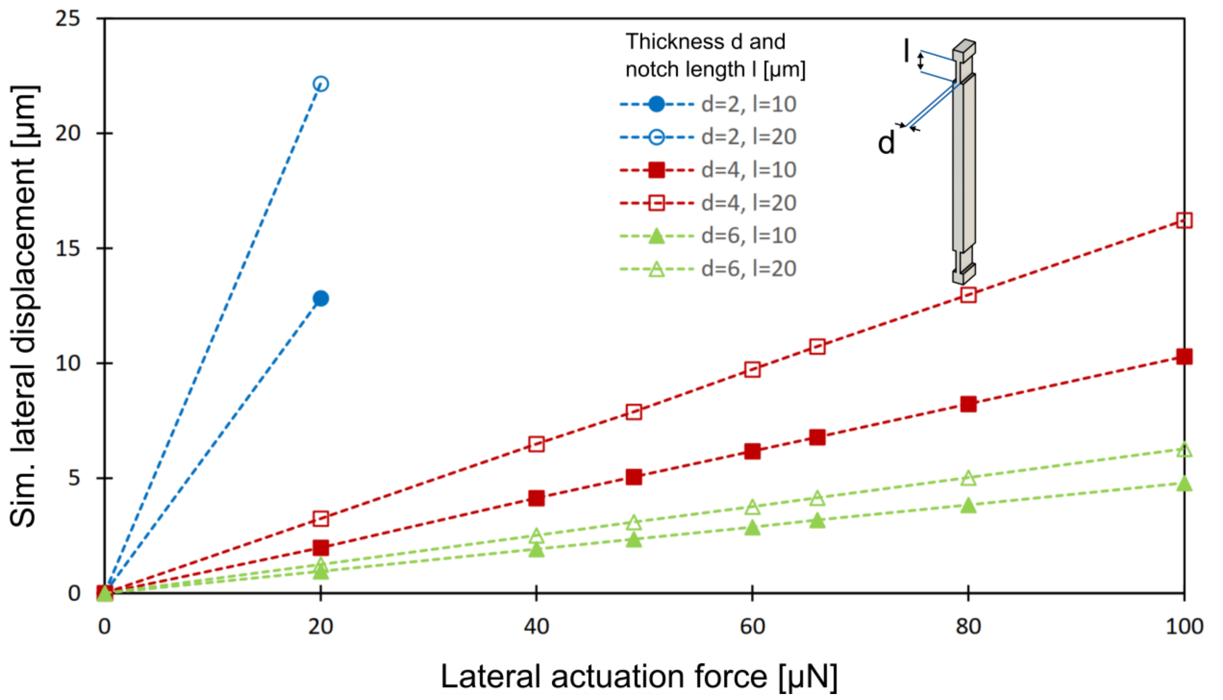

Supplementary Figure S5 Simulated lateral displacement depending on the magnetic force and the dimension of the notches (length and thickness). The total length of the flexure hinge is 220 µm in all cases, i.e. 200 µm distance between the outer edges of the upper and lower notch. Red dashed and unfilled square marker correspond to the final design.

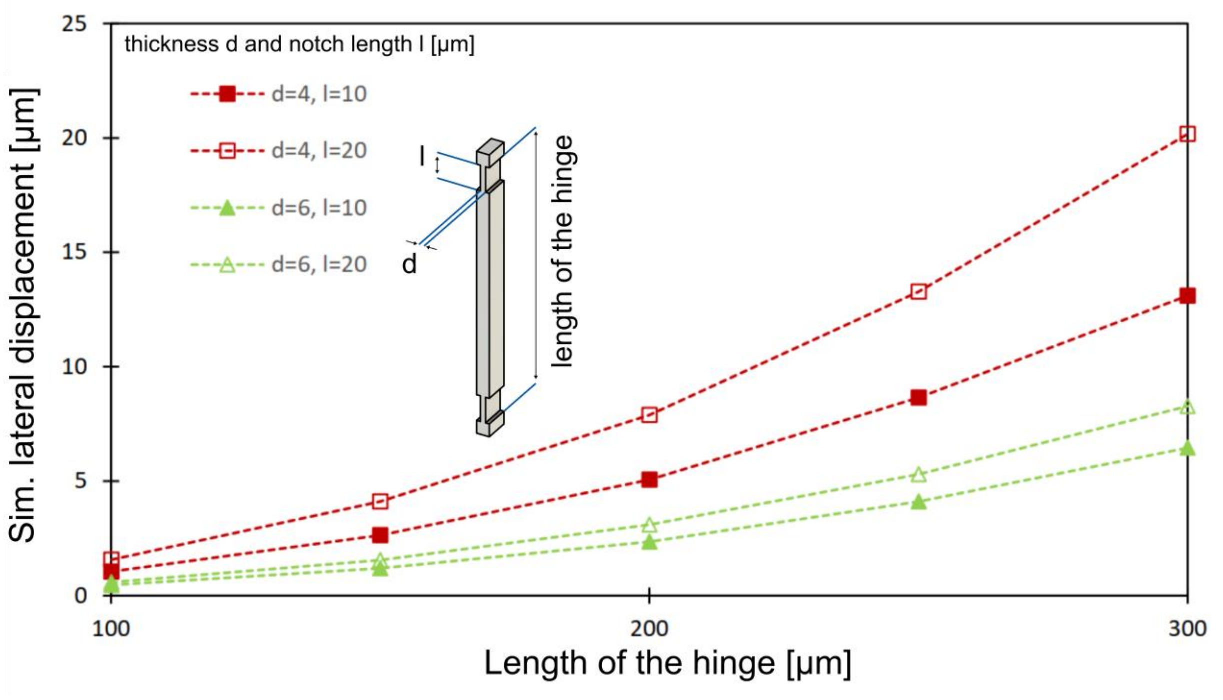

Supplementary Figure S6 Simulated lateral displacement depending on the length of the flexure hinges and the notch design. A lateral magnetic force of 49 µN was assumed, corresponding to a magnet to coil distance of 425 µm and 160 mA. Red dashed and unfilled square marker correspond to the final design.

5. Supplementary Movies

As a general information and as it is stated in the main text, the microsystem and corresponding images were not observed simultaneously. For the videos, the recorded motion of the microsystem and the corresponding change in the image were synchronized.

- Supplementary Movie S1: The video shows the axially actuatable system (Fig. 2) during the actuation experiment alongside the corresponding image observed at the end facet of the fiber bundle.
- Supplementary Movie S2: The video shows the laterally actuatable system (Fig. 3) during the actuation experiment alongside the corresponding image observed at the end facet of the fiber bundle. The playback speed is sped up 4 times.
- Supplementary Movie S3: The video shows the rotatory actuatable system (Fig. 4) during the actuation experiment alongside the corresponding image observed at the end facet of the fiber bundle. Continuous shifting as well as fast switching of the FOV are shown. As fast switching was manually controlled, the videos of the microsystem and the image are not synchronous.
