# Supplementary material for: Magnetically actuatable 3D-printed endoscopic microsystems
Source: Commun Eng. 2025 Apr 9;4:69. doi: 10.1038/s44172-025-00403-8 (PMC11982310; doi:10.1038/s44172-025-00403-8)
Supplement: Supplementary file 2 — Description of Additional Supplementary Files [file 44172_2025_403_MOESM2_ESM.pdf]

Description of Additional Supplementary Files

File name: Supplementary Movie 1

Description: Axially actuatable system

File name: Supplementary Movie 2

Description: Laterally actuatable system (4x playback speed)

File name: Supplementary Movie 3

Description: Rotationally actuatable system
